# Resveratrol as a Promising Nutraceutical: Implications in Gut Microbiota Modulation, Inflammatory Disorders, and Colorectal Cancer

**DOI:** 10.3390/ijms25063370

**Published:** 2024-03-16

**Authors:** Vidhya Prakash, Chinchu Bose, Damu Sunilkumar, Robin Mathew Cherian, Shwetha Susan Thomas, Bipin G. Nair

**Affiliations:** 1 School of Biotechnology, Amrita Vishwa Vidyapeetham, Kollam 690525, Kerala, India; 2 Department of Cancer Biology and Genetics, College of Medicine, The Ohio State University, Columbus, OH 43210, USA

**Keywords:** natural products, food, resveratrol, polyphenols, gut microbiome, cancer

## Abstract

Natural products have been a long-standing source for exploring health-beneficial components from time immemorial. Modern science has had a renewed interest in natural-products-based drug discovery. The quest for new potential secondary metabolites or exploring enhanced activities for existing molecules remains a pertinent topic for research. Resveratrol belongs to the stilbenoid polyphenols group that encompasses two phenol rings linked by ethylene bonds. Several plant species and foods, including grape skin and seeds, are the primary source of this compound. Resveratrol is known to possess potent anti-inflammatory, antiproliferative, and immunoregulatory properties. Among the notable bioactivities associated with resveratrol, its pivotal role in safeguarding the intestinal barrier is highlighted for its capacity to prevent intestinal inflammation and regulate the gut microbiome. A better understanding of how oxidative stress can be controlled using resveratrol and its capability to protect the intestinal barrier from a gut microbiome perspective can shed more light on associated physiological conditions. Additionally, resveratrol exhibits antitumor activity, proving its potential for cancer treatment and prevention. Moreover, cardioprotective, vasorelaxant, phytoestrogenic, and neuroprotective benefits have also been reported. The pharmaceutical industry continues to encounter difficulties administering resveratrol owing to its inadequate bioavailability and poor solubility, which must be addressed simultaneously. This report summarizes the currently available literature unveiling the pharmacological effects of resveratrol.

## 1. Introduction

Resveratrol, a stilbene compound found in grapes, apples, blueberries, plums, and peanuts, is regarded as one of the most extensively researched food components [1]. Plants produce these natural polyphenols as a part of their defense mechanism against pathogens. It is interesting to note that resveratrol is an active food component, imparting more relevance to the quote of Hippocrates: “Let food be thy medicine and medicine be thy food”. The bioactivities and therapeutic potential of resveratrol as a pharmaceutical and nutraceutical compound have been widely investigated ever since it was reported to exhibit strong anticancer properties in 1997 [2]. Resveratrol has been found to have antioxidative, anti-inflammatory, and immunomodulatory properties in several in vitro and in vivo investigations [3]. Promising results from many experimental studies indicate the positive impact of resveratrol supplementation in regulating gut health [4], cardiovascular diseases [5], inflammatory bowel disorders [6], cancer [7], liver diseases [8], diabetes [9], obesity [10], respiratory disorders [11], hypersensitivity [3], Alzheimer’s disease [3], and Parkinson’s disease [3]. These observations make resveratrol a remarkable compound with holistic effects at multiple levels in many diseases. The challenge faced by the scientific community to transform the vast success stories of resveratrol at the laboratory scale into promising outcomes in clinical trials is a significant topic that must be considered. The reduced bioavailability and metabolic vulnerability of resveratrol are implicated as the major drawbacks. There is extensive metabolism of resveratrol by the gut microbiota and phase 2 enzymes, resulting in relatively low levels of free resveratrol reaching the systemic circulation [12]. It should be noted that dosage variations in subjects and metabolic profile differences from individual to individual would explain the lack of positive findings in clinical trials. Even though resveratrol has been extensively researched, resveratrol-derived metabolites such as dihydro-resveratrol, lunularin, oxyresveratrol, pterostilbene, and other resveratrol-conjugated entities have been less explored. A significant portion of the administered resveratrol, which is converted into such forms, could manifest positive biological effects [13,14,15].

Hence it is imperative for future studies to prioritize investigating the indirect activities exhibited by resveratrol through the modulation of microbiota and resveratrol-derived metabolites. Examining the influence of resveratrol on microbiota, elucidating the diverse resveratrol-derived metabolites generated by gut microbiota, and investigating the ways in which these derivatives modulate various diseases could serve to integrate different facets of utilizing resveratrol as a valuable nutraceutical. The inability of free resveratrol to produce significant biological activity at tissue-relevant levels can be resolved through synergy with other compounds. Interestingly, research in different formulation strategies has addressed innovative methods to improve the bioavailability of resveratrol. Hence, we have attempted to summarize the recent findings regarding the influence of resveratrol through microbiota modulation and its resveratrol-derived metabolites in the context of gut health, cancers, and allergic diseases.

## 2. Different Sources of Resveratrol

**Figure 1:**
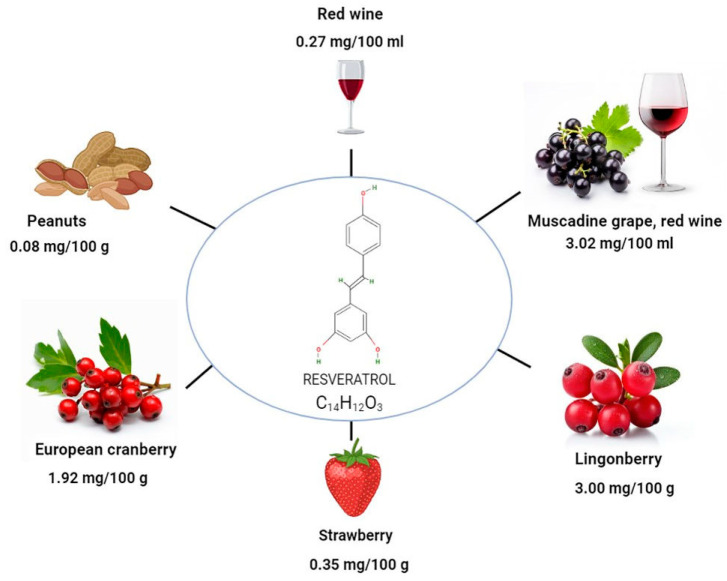
Different sources of resveratrol.

**Figure 2:**
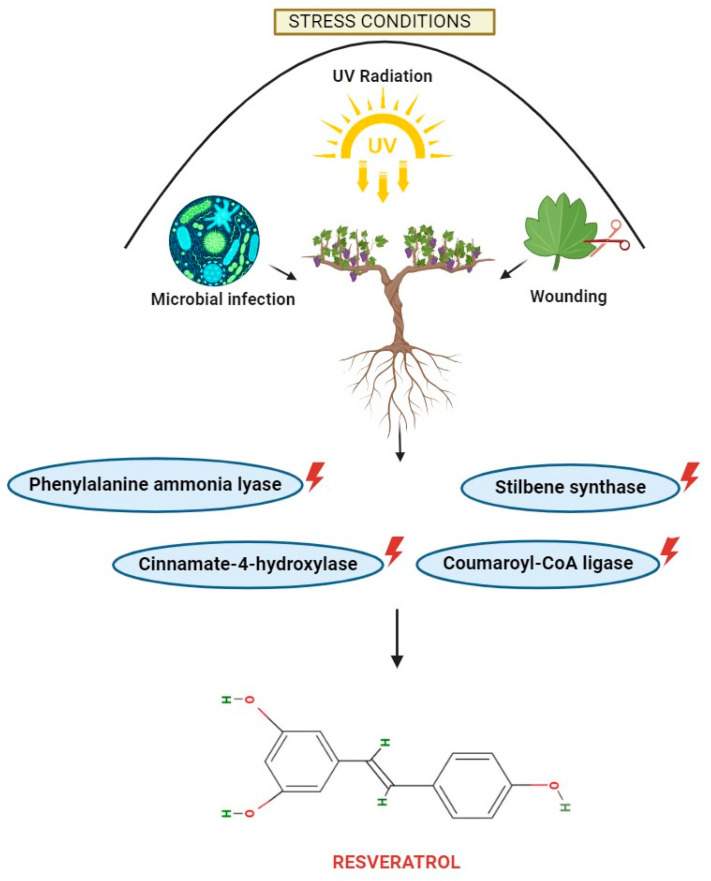
Factors influencing the synthesis of resveratrol. Plants produce resveratrol as a stress response with the aid of enzymes.

The major plant sources of resveratrol, which naturally exists as phytoalexin, are indicated in Figure 1. It is found in the skin, seeds, stems, shoots, buds, roots, and leaves of grapes. Resveratrol accumulation in grapes varies according to the grape cultivar, genotype, location, environmental conditions, and growing season. In comparison with grape juice and wine, grape skin contains a significant amount of the compound [16]. Plants synthesize this compound as an adaptive response to adverse conditions, including exposure to ultraviolet radiation, fungal infections, and physical damage, exemplifying their capacity for sophisticated biochemical adaptation strategies [17,18,19] (Figure 2). The most prevalent antioxidants in our diet are polyphenols, which are also found in cereals, olives, dry beans, licorice, chocolate, and drinks [20]. The flavonoids found in fruits and vegetables belong to the polyphenol class and even contribute to the color of plants [21].

Resveratrol is a polyphenol that has received much attention lately [10,22]. The 1940s saw the first successful isolation of resveratrol by Takaoka from the roots of the toxic plant *Veratrum grandiflorum*, a white hellebore lily [23,24]. Resveratrol extracted from *Polygonum cuspidatum* (Japanese knotweed), is also used in Chinese medicine for therapeutic purposes. The root extracts of field-grown *P. cuspidatum* are currently used to manufacture most of the resveratrol sold on the global market [25]. Recent investigations have also documented the utilization of these compounds as food additives and functional polymer materials, indicating their potential applications in diverse fields. The aromatic amino acids L-phenylalanine (L-Phe) and L-tyrosine (L-Tyr), both of which are produced from the shikimate pathway, are commonly used in the biosynthesis of resveratrol [26]. According to Future Market Insights of 2019, the market is currently worth about USD 97.7 million, and from 2018 to 2028, it is anticipated to develop at a CAGR of 8.1% [27]. While chemical synthesis enables the production of significant quantities of resveratrol, this method is fraught with the generation of numerous undesirable byproducts, complicating the compound’s purification process and posing challenges to its consumption safety. As an alternative biosustainable resource, various biotechnological techniques have been used, including tissue culture and genetic engineering [28].

An intriguing approach with promising potential to address the prevailing challenges in resveratrol manufacturing is the bioproduction of resveratrol through the utilization of recombinant microorganisms [29]. According to Thapa et al., heterologous expression of the plant pathway has been successfully used in several hosts to synthesize resveratrol [30,31]. As higher levels of resveratrol inside cells may cause detrimental effects and reduce resveratrol production, the compound produced within the cells was removed and transferred outside via the four endogenous transport mechanisms of *E. coli* to balance intracellular content and increase production. Zhao et al. explored the effects of various efflux pump proteins, namely, MdfA, EmrD, EmrE, AcrAB, TolC, and MarA, two YddG, AraE, and two OmpW and OmpF. They found that overexpression of MarA and OmpF resulted in the production of 123.16 mg/L and 151.89 mg/L of resveratrol, respectively [32].

**Figure 3:**
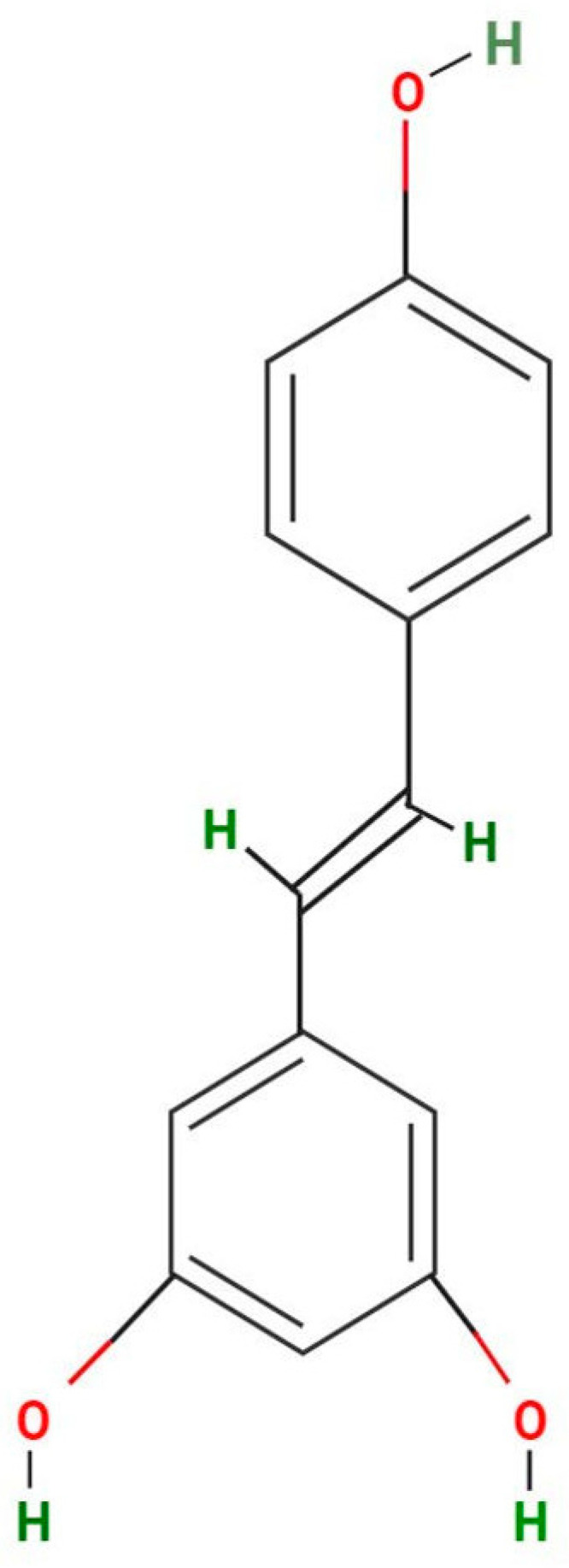
The trans form of resveratrol.

A research study unveiled the presence of resveratrol in tempeh, an Indonesian fermented soybean dish, as well as in the soybean seed coat, which serves as a byproduct of tempeh production. In that study, 65.15% of resveratrol was extracted from tempeh and 55.35% from the soybean seed coat. Furthermore, the study showed that soybean fermentation during tempeh production, prompted by the presence of typical fermented bacteria, resulted in elevated levels of trans-resveratrol [33]. A significant amount of trans-resveratrol accumulation was observed following the pruning of vine shoots, with a maximum induction of 181-fold on day 70. The substantial increase in trans-resveratrol content in vine-shoot samples can be attributed to the structural genes *PAL*, *C4H*, *4CL*, and *STS*, which control trans-resveratrol production. The trans form (Figure 3) is believed to accumulate until the tissues are dry, since living plant tissues may perceive the drying process during the storage period as a stress signal, resulting in the production of this monomer [34].

## 3. Multifaceted Role of Resveratrol in Enhancing Gut-Barrier Integrity, Modulating Microbiota, and Combating Metabolic Disorders

Low molecular stilbenes like resveratrol are preferentially transported to the large intestine, even though a small proportion of the polyphenols found in food are absorbed in the small intestine [35]. Upon metabolism, sulfates, glucuronides, and other conjugated forms of resveratrol exit the intestine via the transporter breast-cancer-resistant protein (BCRP). It has been reported that they can reach the intestinal lumen via multidrug-resistant protein 2 (MRP2) on the apical membrane and enter into the systemic circulation via MRP3 on the basolateral membrane [35]. A substantial portion of ingested resveratrol (approximately 70–75%) undergoes metabolism by intestinal and gut microbiota, with 25% being excreted without absorption, and only 1–8% is detected in the bloodstream [36]. Unmetabolized resveratrol is difficult to detect in blood plasma. Peak concentrations of less than 10 ng/mL were seen within 0.5–2 h following oral administration, based on rough estimates [37,38]. Consuming resveratrol with a meal dramatically reduced its absorption, particularly with high-fat diets [39]. Natural grape products facilitate better resveratrol absorption compared with pills, underscoring the influence of the matrix on bioavailability [40]. Notably, resveratrol delivered in tablet form exhibits prolonged presence in the body, resulting in elevated levels of metabolites [41]. Resveratrol metabolites are of paramount importance as some of them, such as dihydroresveratrol and lunularin have been reported to show potent antioxidative activities [35,42]. It is interesting to note that dihydroresveratrol has been demonstrated to possess more effective antioxidative capabilities than vitamin E [43]. Research has indicated that certain gut microbiota, such as *Bacteroidetes*, *Actinobacteria*, *Verrucomicrobia*, and *Cyanobacteria*, can metabolize resveratrol into 3,4′-dihydroxybibenzyl (lunularin), whereas *Slackia equolifaciens and Adlercreutzia equolifaciens* can produce dihydroxyresveratrol and lunularin [35]. Extensive research has demonstrated that resveratrol and its metabolites impact gut health in a positive manner [44]. Research in mouse models shows that resveratrol supplementation enhances the expression of zonula occludens 1 (ZO-1), zonula occludens 2 (ZO-2), occludin, junctional adhesion molecule A (JAM-A), mucin proteins (MUC 1 and MUC 2), and cathelicidins. These are tight-junction proteins, whose upregulation is known to favorably affect the mucosal barrier [35,45]. Resveratrol supplementation increased the jejunal villus height, thereby improving intestinal morphology, as shown in piglets [46]. Data obtained from research conducted in high-fat diet (HFD)-induced non-alcoholic fatty-liver disease (NAFLD) mice models indicate that resveratrol markedly increased the ileal villus length [45]. These data are important in inferring the impact of resveratrol on intestinal morphology, considering the significance of villi in nutrient absorption and transportation [47]. Changes in gut microbiota composition have been associated with the initiation and advancement of numerous disorders encompassing a spectrum of chronic illnesses [48,49]. Research into how variations in gut microbiota influence human health have gained attention and is one of the immensely researched areas in the current scenario [50,51]. An exploration into microbiota modulation and its ramifications within diseased models offers a highly insightful perspective. Endothelial dysfunction has been characterized by disturbances in nitric oxide (NO) bioavailability, an increase in free radical generation, and increased NLR-family purin-domain-containing 3 (NLRP3) inflammasome activity [52]. Asymmetric dimethylarginine (ADMA) is a competitive inhibitor of NOS and reduces the production of NO. Research conducted in male rats with increased circulatory ADMA levels exacerbated endothelial dysfunction by implementing changes to the gut microbiota composition and intestinal and dorsal hippocampal NLRP3 activation, eventually resulting in cognitive impairment [52]. An increase in microorganisms belonging to the class *Bacteroidia* in ADMA-induced endothelial dysfunction rats is noteworthy as *Bacteroidia* have been linked to chronic social-defeat stress because of its inflammation effects [52]. A reduction in the genus *Anaerotruncus* was also observed in the diseased models. Resveratrol intake was noted to restore the levels of these bacteria back to normal [52]. Based on studies conducted in high-fat-diet-induced NAFLD mice models, the data indicate that resveratrol supplementation ameliorates NAFLD by changing the gut microbiota composition, reducing body weight (BW), improving the gut-barrier integrity, and ameliorating other key features of NAFLD, such as insulin resistance, lipid deposition, oxidative stress, and inflammation [45]. Reduction in the sulfate-reducing bacteria *Desulfovibrio* [45], which can generate lipopolysaccharides (LPS) and induce related inflammation, was observed on resveratrol supplementation. *Alistipes*, whose abundance is correlated with obesity and other metabolic disorders, was also reduced in the HFD-induced NAFLD mice treated with resveratrol [45]. A significant increase in short-chain fatty acid (SCFA)-producing bacteria such as *Allobaculum* and *Blautia* was evident [45]. Similar research demonstrated that resveratrol intake upregulated the concentration of SCFAs such as isobutyric acid and butyrate in the gut [53]. Qiu et al. demonstrated increased abundance of *Roseburia* in the gut of resveratrol-treated piglets [46]. This bacterium has been shown to play a crucial role in preventing pathogenic infection and alleviating intestinal inflammation. Another study reported an increase in *Lactobacillus reuteri*, a well-known probiotic bacterium that can produce antimicrobial molecules, in the colon [53]. The same study reported an increase in bacteria belonging to the phyla *Tenericutes* and *Actinobacteria*, which play important roles in degrading recalcitrant carbon sources in the stomach and protecting against invading viruses, respectively [53]. Resveratrol alleviates CCl_4_-induced liver fibrosis by restraining the growth of *Staphylococcus xylosus* and *Staphylococcus lentus. Staphylococcus*, have been identified as a predominant pathogen in patients with chronic liver diseases, and is often correlated with occurrence of cystic fibrosis [54]. The ability of resveratrol to engage with microorganisms has yielded beneficial outcomes extending beyond gut health. Multiple microorganisms residing in different regions of the body have been demonstrated to interact with resveratrol (Table 1).

## 4. Potential of Resveratrol and the Gut Microbiome in Cancer Prevention

**Figure 4:**
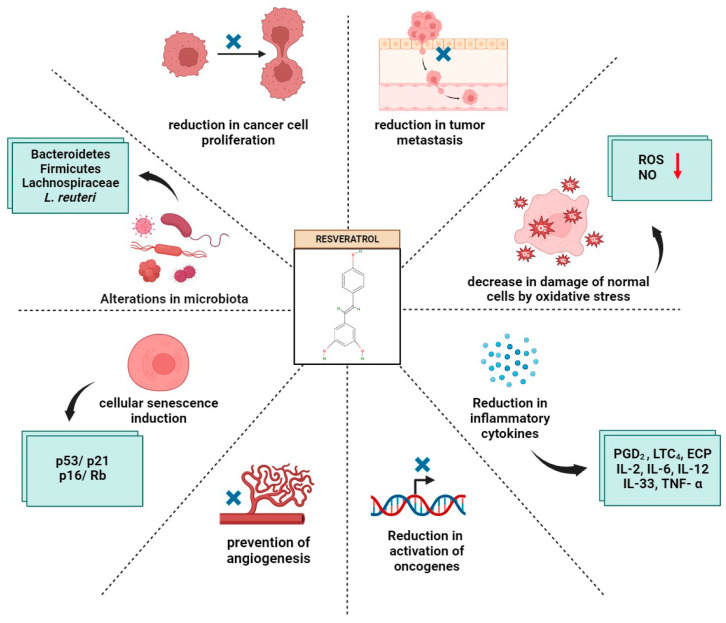
Anticancer effects of resveratrol by modulating different aspects of tumor progression.

Over the past few years, extensive research has been conducted on the effects of resveratrol supplementation on the progression of different cancer types [57,58,59]. Numerous in vivo and in vitro experiments support the anticancer effects of resveratrol; it can inhibit all stages of carcinogenesis, including initiation, promotion, and progression (Figure 4). It also helps to reduce inflammation and protect against oxidative stress, which can damage cells and lead to cancer [59]. Furthermore, the compound reduces inflammatory levels, suppresses new blood-vessel growth in tumors [60], reduces the expression of cancer-causing genes [61], and reduces oxidative stress. Resveratrol also boosts the body’s immune system, making it more likely to fight cancer by improving the immune system. Several studies have demonstrated that resveratrol prevents cancer-cell proliferation, inhibits tumor metastasis, and induces apoptosis [58,60,61,62]. It has been shown that resveratrol interacts with DNA to prevent cancer initiation and progression [62]. Certain bacteria are associated with inflammatory molecules, which may result in inflammation across a wide range of tissues in the body [63]. There has also been extensive research on the relationship between alterations in the composition of the microbiota and their subsequent effects on tumorigenesis. Inflammation, the gut microbiota, and cancer are interconnected. Pancreatic, gastric, colon, liver, breast, and prostate cancer patients have altered microbiota associated with chronic inflammation. Tumorigenesis is promoted by altered gut microbiota by increasing the exposure of gut epithelium to carcinogens, and IRAK-M negative regulation is crucial for colon-cancer resistance [64]. Inflammatory bowel disease (IBD) and colorectal cancer (CRC) patients have drastically altered gut microbiota composition compared with healthy people [65]. Excess oxidative stress, the activation of oncogenes, and negative effects on tumor-suppressor genes have been observed in patients with inflammatory bowel disease [66]. Chronic inflammation of the colon may be one of the causes of colorectal cancer [67]. In hereditary and sporadic CRC cases, anti-inflammatory medications may prevent or delay the development of the disease. CRC risks may be reduced by anti-inflammatory medications, such as non-steroidal anti-inflammatory drugs (NSAIDs) [68]. In addition, the presence of certain bacteria in the colon can further facilitate the growth of these tumors, thereby increasing the risk of colorectal cancer in the colon. IBD and CRC patients can reduce their risk of colorectal cancer by detecting and monitoring their gut microbiota at an early stage. The dysbiosis of gut microbiota, characterized by a restriction of microbial diversity, has been attributed to the rise in colorectal cancer associated with colitis [65]. There has been extensive research on dietary polyphenols such as resveratrol, curcumin, anthocyanins, and ellagic acid as potential chemotherapeutics for colorectal cancer [65]. In mouse models of azoxymethane (AOM) and dextran sulfate sodium (DSS)-induced colitis, 10% polyphenol-rich grape-powder supplementation increased gut bacterial evenness [65]. A comprehensive review of the relationship between the gut microbiota and colitis-associated colorectal cancer (CAC) has been published by Zhao and Jiang et al. [66]. An underappreciated method to control CAC progression may be gut microbiota modulation. The intake of resveratrol increases the amount of Lachnospiraceae in the gut, resulting in an increase in the production of small-chain fatty acids, such as butyrate [65]. Butyrate action in the gut is associated with anti-inflammatory effects that have been linked to the reduction of CRC-related factors as well as the alleviation of DSS-induced colitis in mice [67]. Inflammation-driven cancers are influenced by changes in the gut microbiome that suppress histone deacetylases and stimulate anti-inflammatory T cells [67]. The mechanisms by which the gut microbiota metabolize resveratrol are paramount to understanding its antiproliferative activity. Epigenetic changes can occasionally result in the development and persistence of multiple kinds of cancer. Reports show that resveratrol establishes four hydrogen bonds with the methyl CpG-binding domain (MBD) proteins, as anticipated by molecular docking, and binds with a greater degree of affinity toward the MeCP2 protein (ΔG = −6.5). Investigation of binding energies revealed that the resveratrol in the inhibitor molecule binds to the MeCP2 receptor with a binding energy of −94.76 kJ mol^−1^, MBD2 with a binding energy of −53.83 kJ mol^−1^, and MBD1 with a binding energy of −36.73 kJ mol^−1^ [69]. The therapeutic effects of resveratrol against endometrioid cancer encompass significant oncogenes as well as signaling pathways. To determine potential target genes for resveratrol, the Traditional Chinese Medicine Systems Pharmacology Database and Analysis Platform (TCMSP) was utilized. Studies suggest that the primary interactions between resveratrol and its intended targets include hydrophobic and hydrogen bonding [70]. Using a molecular virtual docker, an in silico approach was conducted to examine the interactions of resveratrol and its analog 3E with proteins such SIRT1, ER, NF-κB, and COX to postulate an effective potential pathway. According to the findings, an effective molecule with higher docking scores for protein interaction is the analog 3E [71]. The docking score value of the phosphodiesterase 4D (PDE4D)–resveratrol complex was investigated using the CANDOCK algorithm and the RMR6 scoring function. It was −30.18, lower than for other polyphenols. The complexes’ structural stability was evaluated using the root-mean-square deviation (RMSD). Because of its compact, firm structure, resveratrol showed a reduced average RMSD value of 0.40 ± 0.04 Å [72]. Reducing the styrene double bond of resveratrol resulted in the production of dibenzyls, which were proven to suppress the growth of tumor cells in colon cancer cells [73]. It is interesting to note that resveratrol does not exhibit significant biological effects in cancer cell types [74]. Gut-derived resveratrol metabolites such as 4-hydroxydibenzyl, lunularin, 3,4′-dihydroxy-trans-stilbene, and dihydroresveratrol attenuated tumor proliferation in human cancer cell lines [74]. At their IC50, these metabolites induced programmed cell death and halted the cell cycle at the S phase. It is also interesting to note that 3,4′-dihydroxy-trans-stilbenes blocked the cell cycle in both the S phase and the G2/M phase [73]. In a similar study, dihydroresveratrol and lunularin, which are derived from the gut microbiota, were found to have chemopreventive effects in renal and colonic cancer cell lines [74]. Several studies have demonstrated that combining dihydroresveratrol with lunularin had strong inhibitory effects on renal and colon cancer cell lines at tissue-relevant levels [74]. These results indicate that gut-derived resveratrol metabolites play a more crucial role than resveratrol itself in mitigating colon cancer. Breast cancer is the first cause of cancer-associated death among women worldwide [75]. Certain resveratrol-conjugate metabolites have been reported to decrease the clonogenic growth of the human breast cancer cell line MCF-7 [76]. Cellular senescence induction was observed in these breast cancer cells with no effect on non-cancerous cells. The resveratrol metabolites modulate senescence induction by regulating the p53/p21 and p16/Rb pathways [76]. It should be noted, however, that the same research also reported dissimilar results regarding MDA-MB-231 cells. Thus, further research is warranted for rectifying the lacunae in this arena. We had earlier established the cytotoxic effects of oxyresveratrol, a hydroxyl-substituted resveratrol derivative, on MDA-MB-231, a highly chemoresistant triple-negative breast cancer cell line [77]. The resolution of such contradictions can only be accomplished through further research. Resveratrol catabolism is a concept that needs to be thoroughly analyzed in the future, particularly in terms of whether it is a deactivation pathway or an activation pathway. It is well known that an individual’s gut microbiota differs in many respects from another’s. Therefore, the various effects observed in different individuals on resveratrol supplementation can be attributed to different resveratrol metabolism metabotypes.

## 5. Anti-Inflammatory and Immunomodulatory Effects of Resveratrol in Asthma and other Inflammatory Diseases

Asthma, a prevalent chronic respiratory condition, exhibits diverse phenotypes linked to gut and lung microbiota dysbiosis [78,79]. Personalized-medicine approaches like microbiome analysis are gaining traction for tailored management [79]. Understanding the interactions between pathogenic and commensal bacteria could illuminate immune dysregulation and inflammation [79]. Asthma is characterized into two divisions: Type 2 and Non-type 2. Paucigranulocytic, neutrophilic, and obesity-related asthma are the Non-type 2 asthmas that are more frequently detected [80]. Resveratrol holds promise for mitigating asthmatic airway inflammation and remodeling [81]. The pathophysiology of asthma is based on an abnormal immunological response to non-pathogenic stimuli in the airways [82]. Inflammation can be brought on by a variety of risk indicators, including microbial invasion or tissue damage [83]. TCM may be created flexibly in accordance with the patient’s physical condition to enable personalized medicinal use with minimum toxicity and adverse effects. It also offers synergistic effects due to its multiple components and targets [84].

Its multifaceted impact involves immunomodulation by suppressing pro-inflammatory cytokine production, enhancing regulatory T cells (Tregs), targeting inflammatory signaling pathways, and reducing reactive oxygen species (ROS) and nitric oxide (NO) levels. Resveratrol diminishes IL-2, IFN-γ, TNF-α, IL-1, IL-6, and IL-17 expression in various immune cells [85,86]. It promotes Treg generation, potentially modulating the gut microbiome through its antimicrobial properties [55]. Type 2 asthma typically responds favorably to glucocorticosteroid therapy [87]. However, there are people with high eosinophilic asthma whose eosinophilia is unresponsive to steroid therapy. Non-type 2 asthma is also associated with poor steroid reactivity [88].

The key glycolipid component of Gram-negative bacterial endotoxins, lipopolysaccharides (LPS), can cause the host to become inflamed [89]. In BV-2 or monocyte LPS-stimulated cells, the addition of resveratrol also resulted in a reduction in the expression of inflammatory mediators such as prostaglandin (PG)E2, COX-2, IL-1, IL-8, TNF-α, and monocyte chemoattractant protein-1 [90,91]. Additionally, resveratrol pre-treatment reduced the expression of the Toll-like receptor-4 (TLR-4) in activated cells and can significantly reduce the levels of IL-6 and TNF-α [92]. Resveratrol can produce anti-inflammatory effects by reducing the levels of ROS and nitric oxide (NO) [93]. Resveratrol controls the inflammatory response through several signaling pathways, including the arachidonic acid, nuclear factor kappa B (NFκ-B) [94], mitogen-activated protein kinase (MAPK) [95], and activator protein-1 (AP-1) [96] pathways.

Resveratrol inhibits the NFκ-B-driven COX-2 signaling pathway that is triggered by LPS stimulation, and this inhibition is related to the activation of AMP-activated kinase (AMPK). Cancerous cells overexpress COX-2 isoenzymes; therefore, the molecular basis for resveratrol’s chemopreventive activity is the compound’s inhibition of COX-2 activity [97]. Treatment with 50 mol/L resveratrol significantly reduced pathological lesions in the rat airways, whereas 10 mol/L resveratrol therapy had little therapeutic impact on the inflammation brought on by asthma [98]. As a result, substantial doses of resveratrol reduced the airway inflammation and remodeling brought on by asthma by preventing the release of inflammatory cytokines via the HMGB1/TLR4/NF-B pathway [98]. By activating sirtuin-1, resveratrol has anti-inflammatory and immunomodulatory properties. By preventing the phosphorylation of p65 and Iκ-B from NFκ-B signaling as well as p38 and ERK from MAPK signaling in mastitis settings, resveratrol can decrease the inflammatory response [99].

Studies suggest that resveratrol alters the gut and lung microbiota composition, potentially impacting asthma development [55]. Resveratrol treatment increases *Akkermansia muciniphila* (anti-inflammatory) and *Bacteroides acidifaciens* (SCFA producer) levels in the gut, potentially favoring Treg generation and reducing LPS levels [55].

Resveratrol inhibits mast-cell activation, crucial in allergic responses, by blocking the MK2/3-PI3K/Akt axis, offering potential for various allergy disorders [100]. In human LAD-2 cells, resveratrol inhibits substance P, compound 48/80, and IgE-mediated degranulation, reducing pro-inflammatory cytokine release [101]. It also downregulates FcεRI (high-affinity IgE receptor) expression, further dampening allergic responses [101]. In humans, resveratrol may be effective in reducing allergic symptoms, particularly allergic rhinitis [102]. It also prevents MC increase in ovalbumin-induced allergic enteritis [101,103]. In addition, it prevents the mitochondrial phosphorylation of extracellular-signal-regulated kinase 1/2 (ERK) along with signal transducer and activator of transcription-3 (STAT-3) to prevent the activation of human intestinal MCs [101,104]. Thus, resveratrol can modulate anti-inflammatory action by downregulating numerous cytokines and their associated signaling pathways in many effector cells (Table 2).

As acute respiratory-distress syndrome is primarily caused by *Staphylococcus* enterotoxin B (SEB), *Lactobacillus reuteri* may be more prevalent in stomachs and lungs exposed to SEB after resveratrol treatment. Resveratrol has been demonstrated to shield *L. reuteri* from protein carbonylation by directly scavenging reactive oxygen species [105]. According to reports, *L. reuteri* can create antimicrobial compounds, preventing the colonization of pathogenic microorganisms. The poor bioavailability poses a huge problem regarding its use as a therapeutic in the context of allergic diseases. Of the total consumed resveratrol, 75% is excreted [102], while the remaining amount undergoes extensive phase-2 metabolism [15,106], catalyzed by different transferase enzymes. Resveratrol in its free form is essential for mediating anti-allergic effects. Thus, other routes of administration should be exploited, or novel strategies of administration should be employed for effective results.

**Figure 5:**
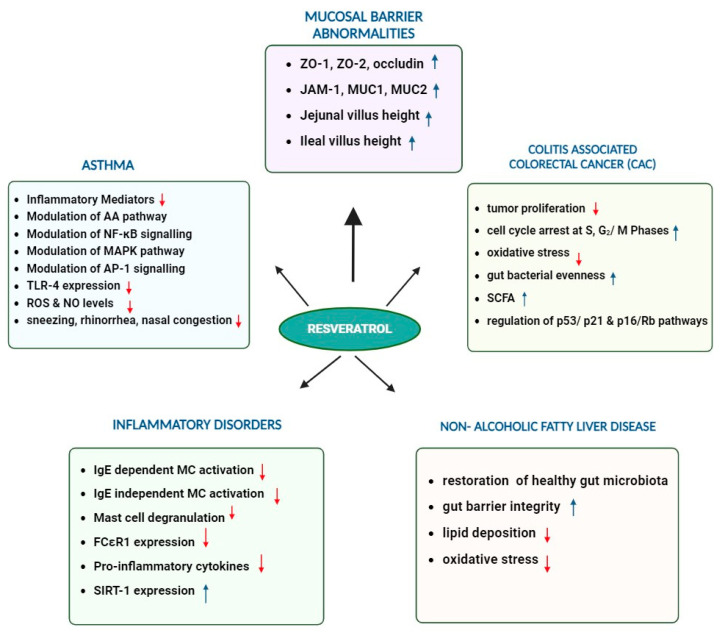
Effect of resveratrol on different disease conditions. Resveratrol supplementation positively influences the downregulation of different disease pathways through various mechanisms (Red arrow: downregulation, blue arrow: upregulation).

A collective summary of the impact of resveratrol through microbiota modulation and resveratrol-derived metabolites in relation to gut health, malignancies, asthma, and inflammatory disorders is presented below (Figure 5).

## 6. Synergistic Effects of Resveratrol with Other Compounds

Several phenolic substances, including antibacterial compounds, have been found in a variety of foods with biological effects. Recent research has demonstrated the effectiveness of plant-derived components in controlling the inflammatory response, which is thought to have health-promoting effects on cellular macromolecules [107]. The biological action of resveratrol can be modified by interacting with these compounds [108]. When used with chemotherapeutic drugs, resveratrol may lessen the adverse effects while increasing the therapeutic effectiveness of cancer treatment. The therapeutic advantages and chemopreventive effects of resveratrol in conditions ranging from autoimmune disorders to neoplastic diseases have recently been reviewed in a few studies [109].

In contrast to Gram-negative bacteria, resveratrol demonstrates significant inhibitory potential against Gram-positive bacteria. The fractional inhibitory-concentration index (FICI) describes the interaction of binary phenolic mixtures with resveratrol. When the FICI index is more than 0.5 but less than 4, it means that resveratrol and phenolic acids interact in additive or neutral ways. Ketones, kaempferol, and rutin had the best outcomes when combined in equimolar amounts with resveratrol, and against *S. aureus*, *B. cereus*, and *E. coli*, resveratrol and kaempferol had a verified synergistic impact (FICI ≤ 0.5), whereas the effect on *S. infantis* was additive. The synergistic action of rutin and resveratrol was demonstrated more evidently against *S. aureus* [56]. A resveratrol and vitamin E combination was free from adverse effects and had specific anti-inflammatory benefits. LPS-induced RAW264.7 cells were treated with low dosages (0.78 g/mL resveratrol + 0.78 g/mL vitamin E) and high dosages (3.13 g/mL resveratrol + 3.13 g/mL vitamin E) to investigate the combined effects of resveratrol and vitamin E. The findings demonstrated that resveratrol and vitamin E combinations resulted in a higher dose-dependent suppression of nitric oxide generation than either agent alone. In LPS-stimulated RAW264.7 cells, resveratrol and vitamin E had a potent anti-inflammatory synergistic effect on oxidative stress and could significantly reduce the transcriptional expression of various inflammatory markers. The combination could further reduce TLR4, p-NF-Bp65, and p-IB signaling-pathway activation in LPS-induced RAW264.7 cells [110]. Our previous studies was able to establish a synbiotic approach in which oxyresveratrol at sub-MIC concentrations could positively modulate the probiotic properties of the strain *Limosilactobacillus fermentum* ASBT-2. Oxyresveratrol was obtained from underutilized coconut shells, establishing an enticing, sustainable approach. The promising synergy between probiotics and polyphenols was clearly established in this study [111].

According to studies, resveratrol improves the DNA-repair capability against clastogenic chemicals by promoting Sirt1 gene expression. Studies also suggest that resveratrol, alpha-lipoic acid, and Q10 can lessen the adverse effects of ionizing radiation on spermatogenesis in mice. The epididymis, basal lamina, and sperm were found to be the targets of these drugs’ most blatant protective actions. These substances, however, were unable to shield the seminiferous tubules and Leydig cells [112]. Multiple pathways related to the cell cycle, apoptosis, and inflammation are modulated by resveratrol [113]. The formation of estrogen–DNA adducts was reduced more in MCF-10F cells treated with resveratrol and N-acetylcysteine concurrently than in cells treated with either chemical alone, and the tumor-initiation process was more effectively inhibited [114]. When resveratrol and ROSC, a CDK inhibitor, were combined to study how they affected the course of the cell cycle in human HL-60 leukemia cells, a startling synergistic impact was observed. In subsequent post-incubation, the proportion of cells in the G1 phase rose to 80% [115]. Resveratrol can prevent or decrease the growth of breast, colon, pancreas, prostate, and liver malignancies, according to recent studies [116,117]. It is also noteworthy that resveratrol, in conjunction with cisplatin, has been used to treat various malignancies, which could reduce cisplatin chemoresistance and side effects [116,118,119].

The most frequent malignant tumor, glioblastoma (GBM), has a dismal prognosis. and the most popular anti-GBM medication is temozolamide [120]. In accordance with data collected from clinics, TMZ is initially effective against GBMs at a rate of about 50%, and secondary medication resistance occurs in about 35% of all instances [120,121]. A type of DNA-repair protein called MGMT is found in a wide range of beings, including bacteria and mammals. It can repair DNA alkylation damage brought on by the drug TMZ, which makes the drug less effective against GBM cells. By reducing the expression of MGMT and downregulating the STAT3/Bcl-2/surviving signaling pathway, resveratrol and temozolomide combination therapy produced synergistic effects that further induced apoptosis and led to cell-cycle arrest in glioma cells [120]. Resveratrol can enhance insulin signaling, lower body weight, and prevent fat storage [122]. Through a SIRT3-dependent mechanism, resveratrol therapy increases insulin sensitivity and reduces body weight [123]. According to Li et al., resveratrol and diet synergistically affect the elevation of SIRT1 levels. Another crucial molecular target for the therapy of resveratrol is AMPK [124,125]. Thus, many compounds have shown synergistic action with resveratrol to treat various complications (Table 3).

## 7. Formulations to Improve the Bioavailability of Resveratrol

The low bioavailability of resveratrol has been attributed to a combination of factors like poor absorption, rapid metabolism, and extensive first-pass elimination, which hinder its potential for therapeutic applications. Considering this, it is crucial to modify and optimize resveratrol to identify improved analogs with significant bioavailability and strong specificities [126,127]. Resveratrol can be made more soluble through encapsulation, protecting it from trans-to-cis isomerization and improving its bioavailability [128]. Liposomes have been used to encapsulate resveratrol within their bilayer structure, protecting it from degradation in the gastrointestinal tract and enhancing its intestinal absorption [128]. A study demonstrated a 4-fold increase in resveratrol bioavailability upon liposomal encapsulation, with peak plasma concentrations reaching 2.8 µg/mL compared with 0.7 µg/mL for the free form [129]. Similar to liposomes, solid lipid nanoparticles (SLNs) offer encapsulation and protection. A 3-fold increase in resveratrol bioavailability has been observed with SLN encapsulation compared with the free form. Peak plasma concentrations reached 1.5 µg/mL with SLNs, compared with 0.5 µg/mL for the free form [130]. The mechanism is thought to involve improved dissolution due to the lipid matrix and potential uptake by intestinal M cells, leading to enhanced lymphatic delivery. Cyclic oligosaccharides form inclusion complexes with resveratrol through non-covalent interactions, increasing its aqueous solubility and dissolution rate, leading to greater contact with the intestinal membrane, which promotes passive diffusion. This leads to improved dissolution in the gastrointestinal tract and enhanced absorption. López-Nicolás et al. has used reversed-phase liquid chromatography to determine the thermodynamic parameters for beta-cyclodextrin complexes of trans-resveratrol [131]. Polymeric nanoparticles, like chitosan and methoxy poly (ethylene glycol)-poly(caprolactone), offer sustained and targeted delivery of resveratrol. These nanoparticles can bypass the first-pass effect and release resveratrol gradually, potentially improving its bioavailability and therapeutic efficacy. Shao et al. incorporated resveratrol into the hydrophobic core of biodegradable nanoparticles made from methoxy poly (ethylene glycol)-poly(caprolactone). The freeze-dried resveratrol-loaded nanoparticles had a diameter of around 90 nm and achieved over 90% encapsulation effectiveness. Resveratrol was released in a steady way from the core-shell nanoparticles, following an initial burst of over 50% in 5 h [132]. Modifying resveratrol’s salt form can influence its solubility and dissolution rate. For example, sodium resveratrolate exhibits higher aqueous solubility compared with the free form, potentially improving absorption. Combining multiple approaches can offer synergistic effects. For instance, resveratrol can also be delivered via blends of emulsions and liposomes. Mixes consisting of glycerol, soybean lecithin, coconut oil, and non-ionic surfactants achieved approximately 70% encapsulation [133]. In conclusion, the pursuit of inventive formulations to improve the bioavailability of resveratrol represents a promising avenue for realizing its potential therapeutic benefits.

## 8. Conclusions

A growing number of studies demonstrate that resveratrol has a profound effect on intestinal tight junctions, microbial composition, and inflammation and has a positive effect on the intestinal barrier, regulating many diseases related to intestinal-barrier damage. Structurally, resveratrol and its derivatives are promising candidates for anti-inflammatory drug formulation. The low bioavailability of resveratrol is believed to be one of the key factors that hinder the translation of the enormous success stories regarding resveratrol at the laboratory scale into promising results in clinical trials. Achieving therapeutic levels of resveratrol from dietary sources alone may be impractical due to its low bioavailability. This raises questions about the appropriate dosage required to achieve the desired health benefits. The supplement industry has responded to the bioavailability issue by producing various resveratrol supplements claiming to provide higher doses. To increase resveratrol’s therapeutic value, it is necessary to improve its bioavailability and activity. Researchers are continuously investigating innovative ways to improve resveratrol delivery into the body, including various formulation techniques and its co-administration with other compounds. The continuous quest to explore the chemodiversity of stilbenes from natural sources is likely to prove promising given the list of new additions of structurally similar compounds with better activity. As we gain a better understanding of resveratrol bioavailability and develop more efficient delivery methods, we might be able to unleash its full potential as a natural health and well-being supplement. More extensive studies would unleash the potential of resveratrol to become an invaluable natural compound for promoting human health.

## Figures and Tables

**Table 1:** Overview of the different microorganisms affected by resveratrol supplementation.

Microorganism	Associated Molecule	Observations/Inferences	Ref.
*Bacteroidetes*, *Actinobacteria*, *Cyanobacteria*	Lunularin	Metabolizes resveratrol to lunularin.	[35]
*Slackia equolifaciens*, *Adlercreutzia equolifaciens*	Dihydroresveratrol, lunularin	Chemopreventive effects in renal and colon cancer cell lines in association with the production of dihydroresveratrol and lunularin.	[35]
*Desulfovibrio*	Resveratrol	Resveratrol supplementation reduced bacterial counts, and downregulated inflammation.	[45]
*Alistipes*	Resveratrol	Decreased abundance of this specific bacterium has been linked to obesity and metabolic disorders.	[45]
*Allobaculum*, *Blautia*	Resveratrol	Elevation of these bacterial levels resulted in enhanced synthesis of SCFAs in the gut.	[45]
*Roseburia*	Resveratrol	Significant increase in levels of this bacteria, which was established to alleviate intestinal inflammation.	[46]
*Anaerotruncus*	Resveratrol	Resveratrol supplementation effectively rescued the bacterial levels to the homeostatic state.	[52]
*Lactobacillus reuteri*	Resveratrol	Significant augmentation of the levels of this well-known probiotic.	[53]
*Staphylococcus xylosus*, *Staphylococcus lentus*	Resveratrol	Reduction in levels resulted in the alleviation of CCl_4_-induced liver fibrosis.	[54]
*Verrucomicrobia*	Lunularin	Metabolic conversion of resveratrol to lunularin.	[55]
*Bacteroides acidifaciens*	Resveratrol	An increase in these bacteria resulted in enhanced production of SCFAs in gut.	[55]
*Staphylococcus aureus*, *Bacillus cereus*, *Escherichia coli*	Resveratrol + kaempferol	The combinatorial supplementation of these compounds demonstrated a synergistic antimicrobial impact.	[56]

**Table 2:** Overview of the different inflammatory cytokines and signaling pathways downregulated by resveratrol.

Cells Involved	Downregulated Molecules/Signaling Pathways	Ref.
Monocytes	PGE2, COX-2, IL-1, IL-8, TNF α, monocyte chemoattractant protein-1 ROS and NO species	[90,91]
Macrophages	IL-2, TNF α	[85]
Lymphocytes	IL-2, IFN γ	[85]
Spleen cells	IL-2, Con A, and allo-antigen-induced proliferation, IL-17 mRNA expression and protein release	[85]
Mast cells	MK2/3-PI3K/AKT signaling, FCεR1 expression	[101,103]

**Table 3:** Overview of different compounds that have shown synergistic action with resveratrol.

Compounds	Observations	Inferences	Ref.
Resveratrol + Kaempferol Resveratrol + Rutin	Inhibited *S. aureus*, *B. cereus*, and *E. coli*	Inhibitory potential against Gram-positive bacteria.	[56]
Resveratrol + Vitamin E	Downregulated TLR4, pN-Bp65, and pIB signaling	Demonstration of anti-inflammatory synergistic effect in murine macrophage cell lines.	[110]
Oxyresveratrol + *L. fermentum ASBT-2*	Inhibited *Salmonella enterica* in the gut	Enhancement of probiotic activity targeting pathogens in the gut.	[111]
Resveratrol + N-acetyl cysteine	Decrease in estrogen–adduct formation	Downregulates adduct formation, which is a crucial factor in breast cancer initiation.	[114]
Resveratrol + ROSC	Increase in cell-cycle arrest of leukemia cells at the G1 phase	Prominent synergistic impact against the cancer of blood-forming tissues.	[115]
Resveratrol + Cisplatin	Effective treatment in many malignancies. Reduction in cisplatin chemoresistance.	A suitable adjunct therapy, which can be considered along with the current standard of care to reduce side effects.	[116,118,119]
Resveratrol + Temozolamide	Lowered MGMT expression. Induction of apoptosis and cell-cycle arrest in glioma cells	An exemplary synergistic combination against glioblastoma.	[120]

## Data Availability

No new data were created or analyzed in this study. Data sharing is not applicable to this article.
